# Neoadjuvant Chemoradiotherapy vs Chemoimmunotherapy for Esophageal Squamous Cell Carcinoma

**DOI:** 10.1001/jamasurg.2025.0220

**Published:** 2025-03-19

**Authors:** Xufeng Guo, Chunji Chen, Jinbo Zhao, Changchun Wang, Xinyu Mei, Jianfei Shen, Huilai Lv, Yongtao Han, Qifeng Wang, Jiahua Lv, Hainan Chen, Xiaolong Yan, Zhichao Liu, Zhengyang Zhang, Qihong Zhong, Youhua Jiang, Liwei Xu, Xiaoyang Li, Dong Qian, Dehua Ma, Minhua Ye, Chunguo Wang, Zimin Wang, Jiangbo Lin, Ziqiang Tian, Xuefeng Leng, Zhigang Li

**Affiliations:** 1 Department of Thoracic Surgery, Shanghai Chest Hospital, Shanghai Jiao Tong University School of Medicine, Shanghai, China; 2 Department of Thoracic Surgery, Tangdu Hospital, Air Force Medical University, Xi’an, China; 3 Department of Thoracic Surgery, Zhejiang Cancer Hospital, Hangzhou, China; 4 Department of Thoracic Surgery, The First Affiliated Hospital of USTC, University of Science and Technology of China, Hefei, China; 5 Division of Life Sciences and Medicine, University of Science and Technology of China, Hefei, China; 6 Department of Thoracic Surgery, Taizhou Hospital of Zhejiang Province affiliated to Wenzhou Medical University, Linhai, China; 7 Key Laboratory of Minimally Invasive Techniques & Rapid Rehabilitation of Digestive System Tumor of Zhejiang Province, Linhai, China; 8 Department of Thoracic Surgery, The Fourth Hospital of Hebei Medical University, Shijiazhuang, China; 9 Department of Thoracic Surgery, Sichuan Cancer Hospital and Research Institute, School of Medicine, University of Electronic Science and Technology of China, Chengdu, China; 10 Department of Radiation Oncology, Sichuan Cancer Hospital and Research Institute, School of Medicine, University of Electronic Science and Technology of China, Chengdu, China; 11 Clinical Research Center for Thoracic Tumors of Fujian Province, Fuzhou, China; 12 Department of Thoracic Surgery, Fujian Medical University Union Hospital, Fuzhou, China; 13 Key Laboratory of Cardio-Thoracic Surgery (Fujian Medical University), Fujian Province University, Fuzhou, China; 14 Department of Radiation Oncology, The First Affiliated Hospital of USTC, University of Science and Technology of China, Hefei, China; 15 EnzeHospital, Taizhou Enze Medical Center, Taizhou, China

## Abstract

**Importance:**

The association of neoadjuvant chemoimmunotherapy (NCIT) vs chemoradiotherapy (NCRT) with tumor downstaging and survival in locally advanced esophageal squamous cell carcinoma (ESCC) remains unclear because of limited evidence.

**Objective:**

To compare the associations of NCIT and NCRT with tumor regression and long-term survival in patients with locally advanced ESCC.

**Design, Setting, and Participants:**

In this comparative effectiveness research study, from January 2016 to March 2023, patients with locally advanced ESCC who underwent esophagectomy following NCRT or NCIT were identified from a prospective database of 8 high-volume esophageal surgery centers in China. Follow-up began on the date of surgery and continued until the last recorded contact or March 2024, whichever occurred first. Data were analyzed between April and September 2024.

**Main Outcomes and Measures:**

The primary end points were 2-year overall survival (OS) and disease-free survival (DFS). Secondary end points included major pathologic response (MPR) and pathologic complete response (pCR). Cox proportional hazard regression analysis was used to investigate the risk factors for OS and DFS.

**Results:**

The study included 1428 patients (median [IQR] age, 63 [57-68] years; 1184 men [82.9%]), with 704 patients in the NCRT group and 724 patients in the NCIT group. After propensity score matching, there were 532 patients in each group. The 2-year OS (81.3% vs 71.3%; hazard ratio, 1.57; 95% CI, 1.26-1.96; *P* < .001) and DFS (73.9% vs 63.4%; hazard ratio, 1.37; 95% CI, 1.11-1.69; *P* < .001) rates were significantly higher in NCIT group than in the NCRT group. The NCRT group had a higher MPR rate than that of the NCIT group (71.8% vs 61.5%), whereas the pCR rates were similar (25.9% vs 22.9%). Multivariable Cox analysis demonstrated that NCIT and MPR were independently associated with both OS and DFS. The NCIT group exhibited a lower overall recurrence rate (126 patients [23.7%] vs 190 patients [35.7%]) and distant metastasis rate (72 patients [13.5%] vs 133 patients [25.0%]), although locoregional metastasis rates were similar (98 patients [18.4%] vs 111 patients [20.9%]). Better OS and DFS were obtained for the NCIT group than for the NCRT group, regardless of whether adjuvant immunotherapy was given.

**Conclusions and Relevance:**

Compared with NCRT, patients with locally advanced ESCC receiving NCIT had better 2-year OS and DFS. The decrease in distant metastasis may be the main reason, but further prospective randomized clinical trials are needed to verify this finding.

## Key Points

**Question:**

Compared with neoadjuvant chemoradiotherapy (NCRT), is neoadjuvant chemoimmunotherapy (NCIT) associated with enhanced tumor regression and long-term survival in patients with locally advanced esophageal squamous cell carcinoma (ESCC)?

**Findings:**

In this comparative effectiveness research study involving 1428 patients in China, the 2-year overall and disease-free survival rates were significantly higher in the NCIT group than in the NCRT group. The major pathologic response rate was higher in the NCRT group than in the NCIT group, but the pathologic complete response rates were similar.

**Meaning:**

These findings suggest that, compared with the current standard NCRT therapy, NCIT is associated with significantly improved long-term survival among patients with locally advanced ESCC.

## Teaser

This comparative effectiveness research study compares the associations of neoadjuvant chemoimmunotherapy and neoadjuvant chemoradiotherapy with tumor regression and long-term survival in patients with locally advanced esophageal squamous cell carcinoma (ESCC).

## Introduction

Neoadjuvant chemoradiotherapy (NCRT) followed by surgery is the current standard care of treatment for locally advanced esophageal cancer.^1,2^ However, one-half of patients encounter disease progression after surgery and typically develop distant metastasis (DM).^3,4^ The JCOG1109 and CIMISG1701 trials confirmed that neoadjuvant chemotherapy (NCT) has efficacy in the treatment of locally advanced esophageal squamous cell carcinoma (ESCC), comparable to that of NCRT, but still faces a high rate of DM after surgery.^5,6^ This suggests that more effective systemic therapy is needed to improve the survival outcomes.

Recent advancements in immunotherapy, particularly immune checkpoint inhibitors (ICIs) targeting the programmed death-1(PD-1)/programmed death-ligand 1 pathway, have shown promising results in advanced esophageal cancer in large randomized clinical trials (RCTs). Immunotherapy combined with chemotherapy has been associated with significantly improved overall survival (OS) compared with conventional chemotherapy as first-line treatment.^7,8,9,10,11^ Consequently, the combination of immunotherapy with chemotherapy has emerged as a potential strategy for locally advanced ESCC. Several clinical trials of neoadjuvant chemoimmunotherapy (NCIT) have reported favorable pathologic complete response (pCR) rates, ranging from 16.7% to 55.6%, with acceptable treatment-related toxic effects.^12,13,14^

Despite these promising findings, whether NCIT yields superior treatment outcomes compared with NCRT warrants further investigation. Recent small-sample retrospective studies suggested that NCIT may offer comparable or even better long-term survival outcomes vs NCRT.^15,16,17^ Here, we present a comprehensive analysis of large-scale, clinical practice data collected from 8 high-volume esophageal surgery centers in China, focusing on patients with locally advanced ESCC who received NCRT or NCIT. This study aims to provide important insights into the optimal neoadjuvant treatment strategy for locally advanced ESCC.

## Methods

This comparative effectiveness research was approved by the Ethics Committee of Shanghai Chest Hospital and adheres to the principles of the Declaration of Helsinki.^18^ Data were obtained from 8 high-volume esophageal surgery centers across China. Because this study is retrospective, informed consent was not required, in accordance with 45 CFR §46.

### Participants

**Figure 1.**
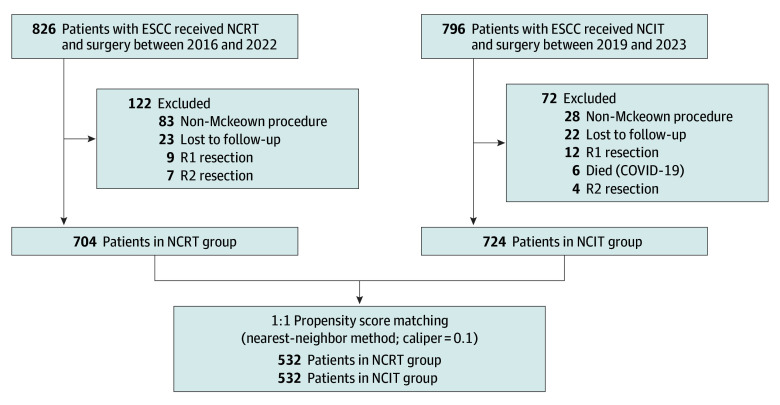
Patient Enrollment Flowchart ESSC indicates esophageal squamous cell carcinoma; NCIT, neoadjuvant chemoimmunotherapy; NCRT, neoadjuvant chemoradiotherapy.

We integrated data from ESCC patient databases from 8 high-volume esophageal surgery centers in China. The NCRT group comprised patients with locally advanced ESCC enrolled between January 2016 and December 2022. The NCIT group comprised patients with locally advanced ESCC enrolled between January 2019 and March 2023. Inclusion criteria were as follows: (1) clinical staging of pretreatment ESCC according to the 8th American Joint Committee on Cancer TNM staging system^19^ as clinical T1b, N1-3, and M0, T2-4a, N0-3, or M0; (2) age 18 to 75 years; (3) normal hematological, kidney, hepatic, cardiac, and pulmonary function; and (4) receipt of NCRT or NCIT. Exclusion criteria included (1) other advanced malignant entities, (2) other types of preoperative treatments, (3) undergoing R1 or R2 resection, and (4) loss to follow-up. The process of patient screening is shown in Figure 1.

### Procedures

In the NCIT group, patients received 2 cycles of ICIs, including pembrolizumab, nivolumab, camrelizumab, sintilimab, toripalimab, or tislelizumab (once every 3 weeks), combined with chemotherapy. For patients who underwent NCRT, neoadjuvant radiotherapy was administered with a total dose ranging from 40.0 to 41.4 Gy, delivered in fractions of 1.8 to 2 Gy, 5 days per week. Both groups received 2 cycles of platinum-based doublet chemotherapy, consisting of a platinum agent (cisplatin, carboplatin, or nedaplatin) combined with paclitaxel or fuorouracil. Adjuvant treatments were then administered on the basis of the pathologic tumor stage and the patient’s recovery condition. Following multidisciplinary discussions, postoperative chemoradiotherapy, chemotherapy alone, or chemoimmunotherapy might be recommended for patients with ypN-positive or ypT1-3N0 disease. In the NCIT group, adjuvant therapy with intravenous PD-1 inhibitors might be recommended for 1 year after surgery.

### Pathologic Examination, Follow-Up, and Outcomes

Pathologic TNM staging was performed according to the 8th American Joint Committee on Cancer staging system. Pathologic samples from each patient were evaluated by 2 experienced pathologists (not coauthors of this article). R0 resection was defined as a curative resection with negative surgical margins (distal, proximal, and circumferential margins). pCR was defined as the absence of viable tumor cells in both the primary tumor site and all dissected lymph nodes. Tumor response to preoperative therapy was assessed using the Chirieac Tumor Regression Grade (TRG) system,^20^ which classifies response into 4 categories: TRG 1, no residual tumor; TRG 2, less than 10% residual tumor; TRG 3, 10% to 50% residual tumor; and TRG 4, more than 50% residual tumor. TRG 1 and 2 are defined as MPR, with less than 10% residual primary tumor, regardless of the lymph node status.

After surgery, patients underwent assessments every 3 months for the first 2 years, followed by every 6 months for the next 3 years, and annually thereafter. Survival status, disease progression, and additional treatments were documented at each follow-up.

### Statistical Analysis

Data were analyzed between April and September 2024. Nearest-neighbor propensity score matching (PSM) was performed in a 1:1, nonreplacement manner with a caliper of 0.1. The balance of baseline covariates before and after matching was evaluated using standardized mean differences (SMDs). An SMD of less than 0.1 was considered to indicate an adequate balance between the groups after matching. Survival outcomes between the 2 groups were compared using Kaplan-Meier curves and the log-rank test. Cox proportional hazards regression was used to assess prognostic factors for OS and disease-free survival (DFS). Data were analyzed with R statistical software version 4.2 (R Project for Statistical Computing) and SPSS statistical software version 21.0 (IBM). A 2-sided *P* < .05 was considered statistically significant.

## Results

### Patient Characteristics

A total of 1428 patients were included in this study (median [IQR] age, 63 [57-68] years; 1184 men [82.9%]), with 704 patients receiving NCRT and 724 patients receiving NCIT. Baseline characteristics are detailed in Table 1. Significant differences between the 2 groups were observed in terms of sex, alcohol misuse, smoking history, clinical T category, clinical N category, and Eastern Cooperative Oncology Group performance status. The NCIT group generally had more advanced clinical T stages (cT3-4a, 649 patients [89.7%] vs 532 patients [75.6%]) and N stages (cN2-3, 236 patients [32.6%] vs 161 patients [22.8%]). To address potential imbalances, PSM was performed. After PSM, there were 532 patients in each group. SMDs of less than 0.10 indicated a well-balanced comparison of baseline characteristics between the groups, as shown in Table 1 and eFigure 1 in Supplement 1.

**Table 1.** Patient Characteristics Before and After Propensity Score Matching

Characteristic	Before propensity score matching				After propensity score matching			
	Patients, No. (%)		*P* value	SMD	Patients, No. (%)		*P* value	SMD
	NCRT (n = 704)	NCIT (n = 724)			NCRT (n = 532)	NCIT (n = 532)		
Age, y								
<60	225 (32.0)	249 (34.4)	.33	0.05	178 (33.5)	172 (32.3)	.70	−0.02
≥60	479 (68.0)	475 (65.6)		−0.05	354 (66.5)	360 (67.7)		0.02
Sex								
Male	606 (86.1)	578 (79.8)	.002	−0.16	443 (83.3)	432 (81.2)	.38	−0.05
Female	98 (13.9)	146 (20.2)		0.16	89 (16.7)	100 (18.8)		0.05
Body mass index^a^								
<18	28 (4.0)	34 (4.7)	.24	0.03	21 (4.0)	22 (4.2)	.75	0.01
18-24	458 (65.0)	440 (60.8)		−0.09	341 (64.1)	329 (61.8)		−0.05
>24	218 (31.0)	250 (34.5)		0.07	170 (31.9)	181 (34.0)		0.04
Smoking history								
No	212 (30.1)	249 (34.4)	.08	0.09	170 (32.0)	194 (36.5)	.12	0.09
Yes	492 (69.2)	475 (65.6)		−0.09	362 (68.0)	338 (63.5)		−0.09
Alcohol misuse								
No	249 (35.4)	213 (29.4)	.02	−0.13	158 (29.7)	178 (33.5)	.19	0.08
Yes	455 (64.6)	511 (70.6)		0.13	374 (70.3)	354 (66.5)		−0.08
Tumor location								
Proximal third	67 (9.5)	90 (12.4)	.08	0.09	55 (10.3)	63 (11.9)	.44	0.05
Middle third	407 (57.8)	429 (59.3)		0.03	316 (59.4)	296 (55.6)		−0.08
Distal third	230 (32.7)	205 (28.3)		−0.10	161 (30.3)	173 (32.5)		0.05
Clinical T category								
cT1	12 (1.7)	11 (1.5)	<.001	−0.02	10 (1.9)	11 (2.1)	.11	0.01
cT2	160 (22.7)	64 (8.8)		−0.49	60 (11.3)	64 (12.0)		0.02
cT3	462 (65.6)	556 (76.8)		0.27	408 (76.7)	378 (71.0)		−0.12
cT4a	70 (10.0)	93 (12.9)		0.09	54 (10.1)	79 (14.9)		0.13
Clinical N category								
cN0	106 (15.1)	108 (14.9)	<.001	−0.004	75 (14.1)	72 (13.5)	.22	−0.02
cN1	437 (62.1)	380 (52.5)		−0.19	312 (58.7)	316 (59.4)		0.02
cN2	153 (21.7)	212 (29.3)		0.17	137 (25.7)	126 (23.7)		−0.05
cN3	8 (1.1)	24 (3.3)		0.12	8 (1.5)	18 (3.4)		0.10
Eastern Cooperative Oncology Group Performance Status								
0	411 (58.4)	355 (49.0)	<.001	−0.19	291 (54.7)	280 (52.6)	.50	−0.04
1	293 (41.6)	369 (51.0)		0.19	241 (45.3)	252 (47.4)		0.04
Received adjuvant therapy								
No	634 (90.1)	313 (43.2)	<.001	NA	472 (88.7)	216 (40.6)	<.001	NA
Yes	70 (9.9)	411 (56.8)		NA	60 (11.3)	316 (59.4)		NA

Abbreviations: NA, not applicable; NCIT, neoadjuvant chemoimmunotherapy; NCRT, neoadjuvant chemoradiotherapy; SMD, standardized mean difference.

^a^ Body mass index is calculated as weight in kilograms divided by height in meters squared.

### Pathologic Examination

The NCRT group demonstrated a significantly higher MPR rate vs the NCIT group (382 patients [71.8%] vs 327 patients [61.5%]), but comparable pCR rates (138 patients [25.9%] vs 122 patients [22.9%]). Primary tumor regression rates were similar between the groups, with comparable rates of TRG 1 (NCIT, 175 patients [32.9%] vs NCRT, 181 patients [34.0%]). No significant differences were observed in lymph node regression between the groups (ypN0, NCIT, 300 patients [56.4%] vs NCRT, 293 patients [55.1%]). The NCIT group achieved a significantly higher lymph node dissection number compared with the NCRT group (median [IQR], 26.0 [19.0-36.0] vs 19.0 [14.0-25.0] lymph nodes), particularly in the thoracic field (median [IQR], 13.0 [8.0-19.0] vs 11.0 [7.0-16.0] lymph nodes) (Table 2). Univariable and multivariable logistic regression analyses indicated that NCIT was independently associated with a lower MPR rate (odds ratio, 0.62; 95% CI, 0.48-0.80; *P* < .001), and was not significantly associated with achieving pCR (odds ratio, 0.85; 95% CI, 0.64-1.12; *P* = .25) (eTable 1 in Supplement 1).

**Table 2.** Pathological Outcomes of Patients Between the NCRT and NCIT Groups Before and After Propensity Score Matching

Variable	Before propensity score matching			After propensity score matching		
	Patients, No. (%)		*P* value	Patients, No. (%)		*P* value
	NCRT (n = 704)	NCIT (n = 724)		NCRT (n = 532)	NCIT (n = 532)	
Lymph node dissection, median (IQR), No.						
Total	19.0 (14.0-25.0)	26.0 (19.0-36.0)	<.001	19.0 (14.0-25.0)	26.0 (19.0-36.0)	<.001
Cervical	0.0 (0.0-0.0)	2.0 (0.0-11.0)	<.001	0.0 (0.0-0.0)	1.0 (0.0-10.0)	<.001
Thoracic	12.0 (8.0-16.0)	13.0 (8.0-20.0)	<.001	11.0 (7.0-16.0)	13.0 (8.0-19.0)	<.001
Abdominal	6.0 (4.0-9.0)	7.0 (0.0-12.0)	.23	6.0 (4.0-9.0)	7.0 (1.0-12.0)	.14
Station 106recR	2.0 (1.0-3.0)	2.0 (1.0-3.3)	.04	2.0 (1.0-3.0)	2.0 (1.0-3.0)	.64
Station 106recL	1.0 (0.0-3.0)	2.0 (0.3-3.0)	<.001	1.0 (0.0-2.0)	2.0 (1.0-3.0)	<.001
Total positive	0.0 (0.0-1.0)	0.0 (0.0-1.3)	.31	0.0 (0.0-1.0)	0.0 (0.0-2.0)	.20
ypT category						
ypT0	255 (36.2)	232 (32.0)	.003	181 (34.0)	175 (32.9)	.08
ypT1	91 (12.9)	143 (19.8)		73 (13.7)	101 (19.0)	
ypT2	97 (13.8)	99 (13.7)		68 (12.8)	73 (13.7)	
ypT3	218 (30.97)	223 (30.8)		176 (33.1)	162 (30.5)	
ypT4	43 (6.1)	27 (3.7)		34 (6.4)	21 (3.9)	
ypN category						
ypN0	377 (53.6)	408 (56.3)	.48	293 (55.1)	300 (56.4)	.98
ypN1	206 (29.3)	185 (25.6)		145 (27.2)	142 (26.7)	
ypN2	94 (13.3)	102 (14.1)		70 (13.2)	67 (12.6)	
ypN3	27 (3.8)	29 (4.0)		24 (4.5)	23 (4.3)	
Chirieac Tumor Regression Grade						
1	255 (36.2)	232 (32.0)	<.001	181 (34.0)	175 (32.9)	<.001
2	263 (37.4)	212 (29.3)		201 (37.8)	152 (28.6)	
3	75 (10.6)	144 (19.9)		61 (11.5)	104 (19.5)	
4	111 (15.8)	136 (18.8)		89 (16.7)	101 (19.0)	
Major pathological response						
No	186 (26.4)	280 (38.7)	<.001	150 (28.2)	205 (38.5)	<.001
Yes	518 (73.6)	444 (61.3)		382 (71.8)	327 (61.5)	
Pathologic complete response						
No	524 (74.4)	560 (77.3)	.20	394 (74.1)	410 (77.1)	.25
Yes	180 (25.6)	164 (22.7)		138 (25.9)	122 (22.9)	
American Joint Committee on Cancer p-staging						
I and II	394 (56.0)	398 (55.0)	.71	306 (57.5)	291 (54.7)	.35
III and IV	310 (44.0)	326 (45.0)		226 (42.5)	241 (45.3)	
Perineural invasion						
Negative	657 (93.3)	644 (89.0)	.004	490 (92.1)	474 (89.1)	.09
Positive	47 (6.7)	80 (11.0)		42 (7.9)	58 (10.9)	
Lymphovascular invasion						
Negative	616 (87.5)	604 (83.4)	.03	454 (85.3)	449 (84.4)	.67
Positive	88 (12.5)	120 (16.6)		78 (14.7)	83 (15.6)	

Abbreviations: NCIT, neoadjuvant chemoimmunotherapy; NCRT, neoadjuvant chemoradiotherapy.

### Postoperative Complications

Overall complication rates were 42.3% (225 patients) in the NCRT group and 37.6% (200 patients) in the NCIT group. The NCIT group showed a significantly higher incidence of pneumonia (22.7% [121 patients] vs 12.2% [65 patients]), pleural effusions requiring drainage treatment (19.6% [104 patients] vs 9.0% [48 patients]), and lower recurrent laryngeal nerve palsy (15.2% [81 patients] vs 19.9% [106 patients]) than those of NCRT group. No significant differences were noted in cardiac complications (NCIT, 20 patients [3.8%] vs NCRT, 13 patients [2.4%]), anastomotic leak (NCIT, 45 patients [8.5%] vs NCRT, 46 patients [8.7%]) and other complications. In addition, both groups had similar reoperation rates and 90-day hospital mortality; each maintained less than 1% without significant differences (eTable 2 in Supplement 1).

### Survival

**Figure 2.**
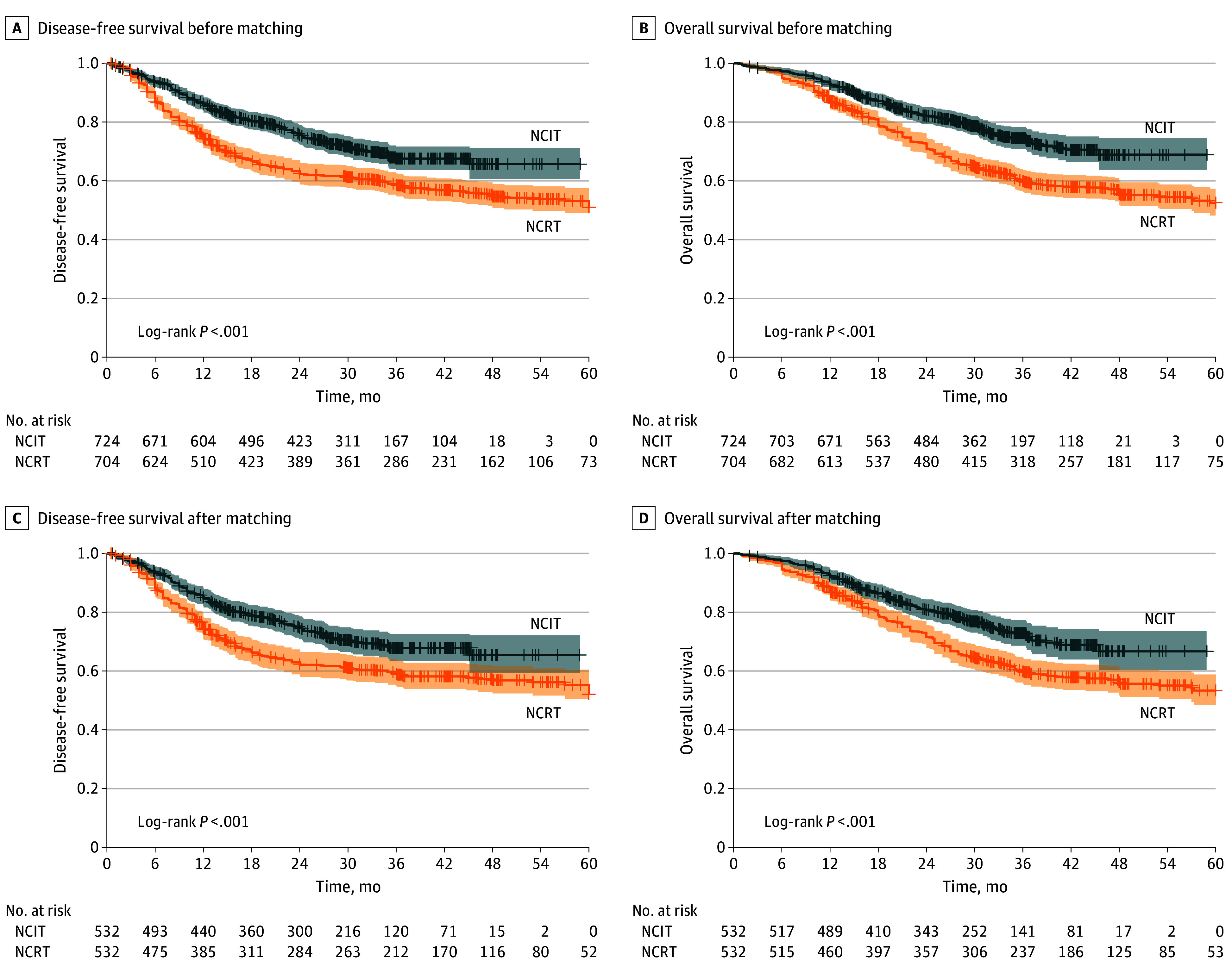
Kaplan-Meier Estimates of Disease-Free Survival and Overall Survival Between Patients Receiving Neoadjuvant Chemoimmunotherapy (NCIT) and Neoadjuvant Chemoradiotherapy (NCRT) Before matching, the 2-year disease-free survival (A) rate for the NCIT group was 75.5% (95% CI, 72.2%-78.8%) vs 62.4% for the NCRT group (95% CI, 58.7%-66.1%) with a hazard ratio (HR) of 1.53 (95% CI, 1.28-1.85). The 2-year overall survival (B) rate for the NCIT group was 82.1% (95% CI, 79.2%-85.0%) vs 71.0% for the NCRT group (95% CI, 67.7%-74.3%) with an HR of 1.67 (95% CI, 1.37-2.03). After matching, the 2-year disease-free survival (C) rate for the NCIT group was 73.9% (95% CI, 69.9%-77.8%) vs 63.4% for the NCRT group (95% CI, 59.1%-67.7%) with an HR of 1.37 (95% CI, 1.11-1.69). The 2-year overall survival (B) rate for the NCIT group was 81.3% (95% CI, 77.8%-84.8%) vs 71.3% for the NCRT group (95% CI, 67.4%-75.2%) with an HR of 1.57 (95% CI, 1.26-1.96).

The median (IQR) follow-up of the survivors was 32.4 (19.8-47.4) months in the NCRT group and 30.2 (19.3-38.4) months in the NCIT group. Among the surviving patients, 816 (84.0%) completed at least 2 years of follow-up. Patients who received NCIT had a significantly higher 2-year OS rate compared with those who received NCRT, both before (594 patients [82.1%] vs 500 patients [71.0%]) and after (433 patients [81.3%] vs 379 patients [71.3%]) matching (hazard ratio [HR], 1.57; 95% CI, 1.26-1.96; *P* < .001). Similarly, the 2-year DFS rate was superior in the NCIT group compared with the NCRT group both before (547 patients [75.5%] vs 439 patients [62.4%]) and after (535 patients [73.9%] vs 446 patients [63.4%]) matching (HR, 1.37; 95% CI, 1.11-1.69; *P* < .001) (Figure 2). Multivariable Cox regression analysis, after matching, confirmed that NCIT was independently associated with both better OS (HR, 0.57; 95% CI, 0.46-0.72; *P* < .001) and DFS (HR, 0.60; 95% CI, 0.48-0.75; *P* < .001) (Table 3).

**Table 3.** Univariable and Multivariable Cox Proportional Hazard Analysis Evaluating Disease-Free and Overall Survival

Variables	Disease-free survival				Overall survival			
	Univariable		Multivariable		Univariable		Multivariable	
	HR (95% CI)	*P* value	HR (95% CI)	*P* value	HR (95% CI)	*P* value	HR (95% CI)	*P* value
Neoadjuvant therapy								
Chemoradiotherapy	1 [Reference]	NA	1 [Reference]	NA	1 [Reference]	NA	1 [Reference]	NA
Chemoimmunotherapy	0.68 (0.55-0.84)	<.001	0.60 (0.48-0.75)	<.001	0.64 (0.52-0.80)	<.001	0.57 (0.46-0.72)	<.001
Age, y								
<60	1 [Reference]	NA	NA	NA	1 [Reference]	NA	NA	NA
≥60	0.84 (0.68-1.05)	.12	NA	NA	0.87 (0.69-1.08)	.20	NA	NA
Sex								
Male	1 [Reference]	NA	NA	NA	1 [Reference]	NA	1 [Reference]	NA
Female	0.82 (0.62-1.08)	.16	NA	NA	0.70 (0.52-0.95)	.02	0.92 (0.64-1.33)	.66
Body mass index^a^								
<18	1 [Reference]	NA	NA	NA	1 [Reference]	NA	NA	NA
18-24	1.63 (0.86-3.08)	.13	NA	NA	1.15 (0.66-2.01)	.62	NA	NA
>18	1.64 (0.86-3.12)	.14	NA	NA	0.99 (0.55-1.76)	.97	NA	NA
Smoking history								
No	1 [Reference]	NA	NA	NA	1 [Reference]	NA	1 [Reference]	NA
Yes	1.21 (0.97-1.51)	.09	NA	NA	1.36 (1.08-1.72)	.01	1.01 (0.70-1.44)	.97
Alcohol misuse								
No	1 [Reference]	NA	NA	NA	1 [Reference]	NA	1 [Reference]	NA
Yes	1.25 (0.99-1.57)	.06	NA	NA	1.34 (1.06-1.70)	.02	1.13 (0.77-1.64)	.53
Tumor location								
Proximal third	1 [Reference]	NA	NA	NA	1 [Reference]	NA	NA	NA
Middle third	0.82 (0.59-1.12)	.21	NA	NA	0.89 (0.64-1.25)	.51	NA	NA
Distal third	0.85 (0.61-1.20)	.37	NA	NA	0.93 (0.64-1.33)	.67	NA	NA
Clinical T category								
cT1	1 [Reference]	NA	NA	NA	1 [Reference]	NA	NA	NA
cT2	1.63 (0.58-4.57)	.36	NA	NA	2.11 (0.65-6.87)	.22	NA	NA
cT3	2.10 (0.78-5.63)	.14	NA	NA	2.77 (0.89-8.64)	.08	NA	NA
cT4	1.91 (0.69-5.33)	.21	NA	NA	2.72 (0.84-8.78)	.10	NA	NA
Clinical N category								
cN0	1 [Reference]	NA	1 [Reference]	NA	1 [Reference]	NA	1 [Reference]	NA
cN1	2.15 (1.45-3.18)	<.001	1.70 (1.13-2.55)	.01	1.91 (1.29-2.84)	.001	1.56 (1.03-2.36)	.04
cN2	2.37 (1.56-3.61)	<.001	1.71 (1.11-2.66)	.02	2.39 (1.57-3.62)	<.001	1.79 (1.15-2.77)	.01
cN3	1.82 (0.83-3.99)	.14	1.75 (0.78-3.93)	.18	2.35 (1.14-4.84)	.021	2.43 (1.14-5.15)	.02
Eastern Cooperative Oncology Group Performance Status								
0	1 [Reference]	NA	NA	NA	1 [Reference]	NA	NA	NA
1	0.92 (0.75-1.14)	.45	NA	NA	1.08 (0.88-1.34)	.47	NA	NA
Major pathological response								
No	1 [Reference]	NA	1 [Reference]	NA	1 [Reference]	NA	1 [Reference]	NA
Yes	0.43 (0.35-0.53)	<.001	0.52 (0.40-0.69)	<.001	0.46 (0.37-0.56)	<.001	0.55 (0.42-0.74)	<.001
Pathologic complete response								
No	1 [Reference]	NA	1 [Reference]	NA	1 [Reference]	NA	1 [Reference]	NA
Yes	0.45 (0.34-0.60)	<.001	1.16 (0.70-1.89)	.57	0.49 (0.36-0.66)	<.001	1.05 (0.64-1.73)	.85
ypT category								
ypT0	1 [Reference]	NA	1 [Reference]	NA	1 [Reference]	NA	1 [Reference]	NA
ypT1	1.60 (1.14-2.25)	.007	1.44 (0.91-2.26)	.12	1.30 (0.91-1.88)	.15	1.17 (0.73-1.87)	.51
ypT2	1.44 (0.99-2.09)	.05	1.00 (0.61-1.63)	.99	1.20 (0.81-1.77)	.37	0.78 (0.47-1.29)	.34
ypT3	2.61 (1.99-3.42)	<.001	1.33 (0.85-2.10)	.21	2.36 (1.80-3.10)	<.001	1.12 (0.71-1.77)	.62
ypT4	2.84 (1.86-4.33)	<.001	1.35 (0.78-2.37)	.29	2.64 (1.72-4.05)	<.001	1.25 (0.71-2.20)	.44
ypN category								
ypN0	1 [Reference]	NA	1 [Reference]	NA	1 [Reference]	NA	1 [Reference]	NA
ypN1	1.79 (1.40-2.28)	<.001	1.49 (1.12-1.99)	.007	1.72 (1.34-2.21)	<.001	1.41 (1.04-1.91)	.03
ypN2	3.05 (2.30-4.05)	<.001	2.37 (1.72-3.27)	<.001	2.88 (2.16-3.84)	<.001	2.02 (1.44-2.83)	<.001
ypN3	3.58 (2.42-5.28)	<.001	1.95 (1.24-3.06)	.004	3.13 (2.07-4.73)	<.001	1.45 (0.90-2.33)	.13
Perineural invasion								
Negative	1 [Reference]	NA	1 [Reference]	NA	1 [Reference]	NA	1 [Reference]	NA
Positive	2.01 (1.48-2.71)	<.001	1.31 (0.94-1.83)	.12	2.13 (1.58-2.88)	<.001	1.39 (1.00-1.95)	.05
Lymphovascular invasion								
Negative	1 [Reference]	NA	1 [Reference]	NA	1 [Reference]	NA	1 [Reference]	NA
Positive	2.04 (1.59-2.62)	<.001	1.20 (0.90-1.60)	.22	2.46 (1.93-3.14)	<.001	1.51 (1.14-2.02)	.005
Received adjuvant therapy								
No	1 [Reference]	NA	NA	NA	1 [Reference]	NA	NA	NA
Yes	0.87 (0.70-1.09)	.22	NA	NA	0.85 (0.67-1.07)	.16	NA	NA

Abbreviations: HR, hazard ratio; NA, not applicable.

^a^ Body mass index is calculated as weight in kilograms divided by height in meters squared.

Multivariable analysis highlighted cN-positive, MPR, and ypN-positive status as being independently associated with both OS and DFS, with cN-positive and ypN-positive being associated with worse survival and MPR associated with better survival. However, pCR was not independently associated with survival in the multivariable analysis (Table 3). To further explore whether MPR and pCR achieved by NCRT or NCIT were similarly associated with outcomes, subgroup analyses were conducted. The results indicated that, among all patients, or those who achieved MPR (eFigure 2 in Supplement 1) or non-pCR (eFigure 3 in Supplement 1), those in the NCIT group had significantly better OS and DFS than those in the NCRT group, regardless of matching status. However, for patients who achieved pCR, there were no statistically significant differences in OS or DFS between the 2 groups, either before or after matching (eFigure 3 in Supplement 1).

In addition, we assessed the 2-year local recurrence (LR)–free survival between the 2 groups. The LR-free survival rates were 84.7% (95% CI, 79.9%-89.4%) in the NCIT group and 83.6% (95% CI, 78.9%-88.3%) in the NCRT group after matching (*P* = .23). Furthermore, the 2-year DM-free survival significantly favored the NCIT group, with rates of 86.5% (95% CI, 83.5%-89.4%) compared with 74.8% (95% CI, 71.4%-78.1%) in the NCRT group after matching (*P* < .001) (eFigure 4 in Supplement 1).

After matching, better OS and DFS were obtained in the NCIT group than in NCRT group, regardless of whether adjuvant immunotherapy was given (316 patients in the NCIT group [59.4%] vs 60 patients in the NCRT group [11.3%] received adjuvant therapy). Nevertheless, there was no statistically significant difference for OS and DFS between the NCIT plus ICI group and the NCIT group (eFigure 5 in Supplement 1).

### Recurrence Pattern

The recurrence rate within 2 years was significantly higher in the NCRT group compared with that in the NCIT group (190 patients [35.7%] vs 126 patients [23.7%]). In the NCRT group, the DM rate was higher than in the LR rate (133 patients [25.0%] vs 111 patients [20.9%]), whereas in the NCIT group, the DM rate was lower than the LR rate (72 patients [13.5%] vs 98 patients [18.4%]). There was no significant difference between the 2 groups in terms of LR rate (NCIT 18.4% vs NCRT 20.9%), including specific patterns such as locoregional lymph node recurrence, anastomotic recurrence, or combined LR. However, the DM rate was significantly higher in the NCRT group compared with that in the NCIT group (25.0% vs 13.5%). Specifically, the NCIT group exhibited a significantly lower incidence of bone (15 patients [2.8%] vs 45 patients [8.5%]) and lung (26 patients [4.9%] vs 60 patients [11.3%]) metastases compared with those in the NCRT group after matching (eTable 3 in Supplement 1).

We further compared recurrence patterns between MPR and non-MPR subgroups in the NCRT and NCIT groups. The findings demonstrated that NCIT was associated with a reduced risk of DM in both MPR (NCIT, 25 patients [7.6%] vs NCRT, 76 patients [19.9%]) and non-MPR patients (NCIT, 47 patients [22.9%] vs NCRT, 57 patients [38.0%]) (eTable 4 in Supplement 1). Similarly, in both pCR and non-pCR subgroups, NCIT also was associated with reduced DM (pCR, NCIT, 6 patients [4.9%] vs NCRT, 18 patients [13.0%]; non-pCR, NCIT, 66 patients [16.1%] vs NCRT, 115 patients [29.2%]) (eTable 5 in Supplement 1).

## Discussion

To the best of our knowledge, this comparative effectiveness research study is the largest-sample, multicenter study that compared NCIT with NCRT for patients with locally advanced ESCC. The results confirmed that NCIT was associated with better 2-year OS and DFS than NCRT, although patients receiving NCRT obtained higher MPR rate and similar pCR rate compared with NCIT. NCIT also showed a favorable control of total recurrence and DM without increased locoregional recurrence within postoperative 2 years.

Both the CROSS trial^3^ and NEOCRTEC_50_10 trial^4^ had confirmed the therapeutic advantages of NCRT over surgery alone for locally advanced ESCC. However, approximately 40% and 30% of patients experienced recurrence and death within 2 years after surgery, respectively. Notably, distant failures remain the main problem of NCRT, which has significantly exceeded the LR (22.0% vs 5.9% in CROSS; 21.7% vs 12.2% in NEOCRTEC_50_10).^3,4^ In addition, JCOG1109^5^ and CIMISG1701^6^ studies confirmed that NCT was not inferior to NCRT with regard to OS, but it also cannot solve the problem of DM very well, suggesting that more effective systemic therapy is needed to further improve the therapeutic effect. In recent years, a number of studies have reported that immunodrugs represented by PD-1 combined with chemotherapy have achieved good outcomes as the first-line treatment for advanced esophageal cancer,^7,8,9,10,11^ and are expected to play a greater role in neoadjuvant therapy. Several phase 2 clinical trials of neoadjuvant immunotherapy demonstrated favorable tumor regression with acceptable treatment related toxicity.^12,13,14^ Then, RCTs were conducted. The ESCORT-NEO trial^21^ was the first phase 3 study worldwide to compare perioperative results of NCIT with NCT, with NCIT achieving higher pCR (28.0% vs 4.7%) and MPR (59.1% vs 20.9%) rates. Treatment-related adverse events were comparable in NCIT and NCT groups (grade ≥3, 34.1% vs 28.8%).^22^ The other RCT also showed that NCIT group had a higher pCR rate (18.6% vs 4.6%) and equivalent treatment-related adverse events (12.5% vs 12.4%) compared with NCT.^22^

In view of this, whether NCIT can achieve better therapeutic effects than NCRT, which is the standard care of treatment of locally advanced ESSC, needs to be answered. Recently published small-sample retrospective studies suggested that NCIT can achieve better OS than NCRT without increasing postoperative complications. Yu et al^15^ reported that NCIT exhibited not only a better 3-year OS than NCRT (91.7% vs 79.8%; *P* = .03), but also a better 3-year DFS (87.4% vs 72.8%; *P* = .04). Yang and colleagues^16^ reported that NCIT was associated with better progression-free survival (HR, 0.50; 95% CI, 0.32-0.77; *P* = .002). However, Wang et al^17^ conducted a single-center retrospective study and reported that, although the pCR was improved in NCRT group compared with NCIT group, there was no significant difference in 3-year DFS and OS between the 2 groups (DFS, 58% vs 56%; OS, 70% vs 72%). The NICE2 study^23^ and Keystone 002 study^24^ are ongoing phase 3 RCTs comparing NCIT with NCRT in locally advanced ESCC, but it will be a long time before the final results are published. Thus, our study provides important insights into the efficacy of these 2 treatment modalities, with important implications for clinical practice based on the largest-sample multicenter clinical practice data up to now.

Our study revealed that NCIT demonstrated a significantly higher 2-year OS (81.3% vs 71.3%) and DFS (73.9% vs 63.4%) compared with NCRT. The results of multivariable analysis suggested that NCIT was a factor independently associated with improved prognosis. These findings align with recent literature indicating the potential benefit of incorporating immunotherapy into the neoadjuvant setting for ESCC.^15,16,22,25^ MPR was also a factor associated with improved survival, but pCR was not independently associated with better survival outcomes. The role of pathologic response, MPR and pCR, as a surrogate for survival in ESCC has been an important topic of ongoing debate. In this study, the pCR rates were similar between the NCIT and NCRT groups (22.9% vs 25.9%), but NCRT demonstrated a significantly higher MPR rate (71.8% vs 61.5%). It might be due to the direct antitumor effect of radiation on the primary tumor. This suggests that although pCR may be a useful short-term indicator of treatment efficacy, it may not be a reliable surrogate for long-term outcomes, particularly when comparing NCRT and NCIT. Our finding is consistent with the results of other studies, which have reported that although pCR is associated with improved survival, it is not always an independent prognostic factor.^15,16,25,26,27^ Interestingly, our subgroup analysis revealed that both patients who achieved MPR and non-MPR in the NCIT group had better OS and DFS than those in the NCRT group.

The divergent survival outcomes observed in MPR and non-MPR patients between the 2 groups suggested that neoadjuvant immunotherapy may play a critical role in determining long-term prognosis. Therefore, it is possible that MPR achieved after NCIT may have a different biological significance compared with MPR achieved after NCRT. Consistent with our findings, several prior studies have also demonstrated that MPR was correlated with enhanced survival rates in non–small cell lung cancer, suggesting that MPR may serve as a viable alternative indicator for OS.^28,29,30,31^ Nevertheless, there has no research regarding the potential use of MPR as a surrogate marker for OS in patients with ESCC who have undergone neoadjuvant therapy in the context of NCIT. Further research is required to clarify this distinction.

In addition, 59.4% of patients in the NCIT group in this study received adjuvant immunotherapy, whereas only 11.3% of patients in the NCRT group received adjuvant therapy. Thus, there is a question that cannot be avoided: was the higher OS in the NCIT group due to adjuvant immunotherapy? To answer this question, we also conducted a subgroup analysis, and the results suggested that there was no significant difference in OS and DFS between NCIT group and NCIT with adjuvant immunotherapy group. Nevertheless, both NCIT subgroups have higher OS than those of the NCRT group. Multivariable analysis also suggested that NCIT but not adjuvant therapy was independently associated with good prognosis. Here, we need to clarify that most of the patients receiving NCRT included in this study received therapy before the publication of CheckMate 577 study,^32^ when most of the patients did not receive adjuvant immunotherapy. Therefore, whether there was a difference in long-term survival between NCIT combined with adjuvant immunotherapy and NCRT with adjuvant immunotherapy still needs to be answered by further studies.

### Limitations

There are several limitations in this study. First, this study was retrospective, but confounding factors were well balanced by PSM. Second, the data came from different centers, and there was a possibility of selective bias in surgical quality control. However, the centers participating in this study were all high-volume centers, which minimized the impact of differences in surgical quality control on survival. Third, patients received different kinds of immunodrugs and different chemotherapy regimens. Fourth, the study object was ESCC, and the research conclusions cannot be fully applicable to esophageal adenocarcinoma.

## Conclusions

In conclusion, this study demonstrated that NCIT is associated with higher 2-year OS and DFS compared with NCRT in patients with locally advanced ESCC, primarily due to its ability to reduce the incidence of recurrence, especially for DM. MPR was a factor independently associated with prognosis, and its significance may differ between NCIT and NCRT, with MPR achieved after NCIT potentially conferring a distinct survival benefit. Future studies are needed to validate these findings and to further elucidate the mechanisms underlying the differential effects of NCIT and NCRT on tumor biology and immune modulation.

## Notes

Supplement 1. **eTable 1.** Univariable and multivariable logistic regression analysis for evaluating MPR and pCR of tumor **eTable 2.** Postoperative complications between the NCRT group and the NCIT group before and after propensity score matching **eTable 3.** Recurrence patterns of postoperative two years between the NCRT group and the NCIT group before and after propensity score matching **eTable 4.** Recurrence patterns of MPR and Non-MPR between the NCRT group and the NCIT group before and after propensity score matching **eTable 5.** Recurrence patterns of pCR and Non-pCR between the NCRT group and the NCIT group before and after propensity score matching **eFigure 1.** A scatter plot of the absolute mean differences in baseline data between the NCRT and NCIT groups before and after matching **eFigure 2.** Subgroup analysis of disease-free survival and overall survival were conducted between the NCIT and NCRT groups, stratified by MPR or Non-MPR **eFigure 3.** Subgroup analysis of disease-free survival and overall survival were conducted between the NCIT and NCRT groups, stratified by pCR or Non-pCR **eFigure 4.** Kaplan-Meier estimates of locoregional relapse-free survival (LRFS) and distant metastasis-free survival (DMFS) between NCIT and NCRT **eFigure 5.** Subgroup analyses of disease-free survival and overall survival between the NCRT group and NCIT group (stratified by adjuvant immunotherapy)

Supplement 2.
Data Sharing Statement
