# Supplementary material for: Neoadjuvant Chemoradiotherapy vs Chemoimmunotherapy for Esophageal Squamous Cell Carcinoma
Source: JAMA Surg. 2025 Mar 19;160(5):565–74. doi: 10.1001/jamasurg.2025.0220 (PMC11923775; doi:10.1001/jamasurg.2025.0220)
Supplement: Supplement 1. — eTable 1. Univariable and multivariable logistic regression analysis for evaluating MPR and pCR of tumor eTable 2. Postoperative complications between the NCRT group and the NCIT group before and after propensity score matching eTable 3. Recurrence patterns of postoperative two years between the NCRT group and the NCIT group before and after propensity score matching eTable 4. Recurrence patterns of MPR and Non-MPR between the NCRT group and the NCIT group before and after propensity score matching eTable 5. Recurrence patterns of pCR and Non-pCR between the NCRT group and the NCIT group before and after propensity score matching eFigure 1. A scatter plot of the absolute mean differences in baseline data between the NCRT and NCIT groups before and after matching eFigure 2. Subgroup analysis of disease-free survival and overall survival were conducted between the NCIT and NCRT groups, stratified by MPR or Non-MPR eFigure 3. Subgroup analysis of disease-free survival and overall survival were conducted between the NCIT and NCRT groups, stratified by pCR or Non-pCR eFigure 4. Kaplan-Meier estimates of locoregional relapse-free survival (LRFS) and distant metastasis-free survival (DMFS) between NCIT and NCRT eFigure 5. Subgroup analyses of disease-free survival and overall survival between the NCRT group and NCIT group (stratified by adjuvant immunotherapy) [file jamasurg-e250220-s001.pdf]

Supplemental Online Content

Guo X, Chen C, Zhao J, et al. Neoadjuvant chemoradiotherapy vs chemoimmunotherapy for esophageal squamous cell carcinoma. *JAMA Surg*. Published online March 19, 2025. doi:10.1001/jamasurg.2025.0220

eTable 1. Univariable and multivariable logistic regression analysis for evaluating MPR and pCR of tumor

eTable 2. Postoperative complications between the NCRT group and the NCIT group before and after propensity score matching

eTable 3. Recurrence patterns of postoperative two years between the NCRT group and the NCIT group before and after propensity score matching

eTable 4. Recurrence patterns of MPR and Non-MPR between the NCRT group and the NCIT group before and after propensity score matching

eTable 5. Recurrence patterns of pCR and Non-pCR between the NCRT group and the NCIT group before and after propensity score matching

eFigure 1. A scatter plot of the absolute mean differences in baseline data between the NCRT and NCIT groups before and after matching

eFigure 2. Subgroup analysis of disease-free survival and overall survival were conducted between the NCIT and NCRT groups, stratified by MPR or Non-MPR

eFigure 3. Subgroup analysis of disease-free survival and overall survival were conducted between the NCIT and NCRT groups, stratified by pCR or Non-pCR

eFigure 4. Kaplan-Meier estimates of locoregional relapse-free survival (LRFS) and distant metastasis-free survival (DMFS) between NCIT and NCRT

eFigure 5, Subgroup analyses of disease-free survival and overall survival between the NCRT group and NCIT group (stratified by adjuvant immunotherapy)

This supplemental material has been provided by the authors to give readers additional information about their work.

© 2025 Guo X et al. *JAMA Surgery.*

eTable 1 Univariable and multivariable logistic regression analysis for evaluating MPR and pCR of tumor.

Variables	MPR				pCR			
	Univariate		Multivariate		Univariate		Multivariate	
	P	OR (95%CI)	*P*	OR (95%CI)	*P*	OR (95%CI)	*P*	OR (95%CI)
Neoadjuvant Therapy								
NCRT		1.00 (Reference)		1.00 (Reference)		1.00 (Reference)		
NCIT	**<.001**	0.63 (0.48 - 0.81)	**<.001**	0.62 (0.48 - 0.80)	0.25	0.85 (0.64 - 1.12)		
Age								
<60		1.00 (Reference)				1.00 (Reference)		
⩾60	0.39	1.13 (0.86 - 1.47)			0.32	1.16 (0.86 - 1.58)		
Gender								
Male		1.00 (Reference)		1.00 (Reference)		1.00 (Reference)		
Female	**0.01**	1.59 (1.11 - 2.27)	**0.008**	1.62 (1.14 - 2.32)	0.15	1.30 (0.91 - 1.85)		
BMI								
<18		1.00 (Reference)				1.00 (Reference)		
18-24	0.62	0.85 (0.43 - 1.65)			0.66	0.86 (0.43 - 1.71)		
>18	0.74	0.89 (0.45 - 1.77)			0.48	0.77 (0.38 - 1.58)		
Smoking history								
No		1.00 (Reference)				1.00 (Reference)		
Yes	0.38	0.89 (0.68 - 1.16)			0.05	0.75 (0.56 - 1.00)		
Alcohol abuse								
No		1.00 (Reference)				1.00 (Reference)		1.00 (Reference)
Yes	0.16	0.82 (0.62 - 1.08)			**0.02**	0.71 (0.53 - 0.95)	0.06	0.75 (0.56 - 1.02)
Tumor location								
Proximal third		1.00 (Reference)				1.00 (Reference)		
Middle third	0.65	0.91 (0.59 - 1.39)			0.84	0.95 (0.60 - 1.52)		
Distal third	0.62	0.89 (0.57 - 1.40)			0.42	1.22 (0.75 - 1.99)		
Clinical T category								
cT1		1.00 (Reference)				1.00 (Reference)		
cT2	0.84	0.89 (0.27 - 2.86)			0.74	1.19 (0.43 - 3.30)		
cT3	0.12	0.42 (0.14 - 1.26)			0.62	0.79 (0.30 - 2.05)		
cT4	0.23	0.49 (0.16 - 1.55)			0.35	0.61 (0.21 - 1.72)		
Clinical N category								
cN0		1.00 (Reference)				1.00 (Reference)		1.00 (Reference)
cN1	0.81	1.05 (0.72 - 1.53)			**<.001**	0.49 (0.34 - 0.72)	**<.001**	0.51 (0.35 - 0.75)
cN2	0.73	0.93 (0.61 - 1.42)			**<.001**	0.44 (0.28 - 0.69)	**<.001**	0.46 (0.30 - 0.73)
cN3	0.07	2.84 (0.93 - 8.68)			0.31	0.62 (0.24 - 1.56)	0.37	0.65 (0.26 - 1.66)
ECOG-PS								
0		1.00 (Reference)				1.00 (Reference)		
1	0.65	0.94 (0.73 - 1.22)			0.62	0.93 (0.70 - 1.23)		

© 2025 Guo X et al. *JAMA Surgery.*

eTable 2. Postoperative complications between the NCRT group and the NCIT group before and after propensity score matching.

Complication category	Before PSM			After PSM		
	NCRT (n = 704)	NCIT (n = 724)	*P* Value	NCRT (n = 532)	NCIT (n = 532)	*P* Value
Overall complication			0.39			0.12
No	412 (58.5)	440 (60.8)		307 (57.7)	332 (62.4)	
Yes	292 (41.5)	284 (39.2)		225 (42.3)	200 (37.6)	
Respiratory complication, n(%)			0.36			1.0
No	515 (73.2)	514 (71.0)		390 (73.3)	390 (73.3)	
Yes	189 (26.8)	210 (29.0)		142 (26.7)	142 (26.7)	
Pneumonia, n(%)			**<.001**			**<.001**
No	630 (89.5)	550 (76.0)		467 (87.8)	411 (77.3)	
Yes	74 (10.5)	174 (24.0)		65 (12.2)	121 (22.7)	
Respiratory failure, n(%)			0.49			1.0
No	692 (98.3)	708 (97.8)		521 (97.9)	521 (97.9)	
Yes	12 (1.7)	16 (2.2)		11 (2.1)	11 (2.1)	
ARDS, n(%)			0.47			0.76
No	698 (99.1)	715 (98.8)		527 (99.1)	526 (98.9)	
Yes	6 (0.9)	9 (1.2)		5 (0.9)	6 (1.1)	
The effusion requires treatment, n(%)			**<.001**			**<.001**
No	646 (91.8)	561 (77.5)		484 (91.0)	428 (80.4)	
Yes	58 (8.2)	163 (22.5)		48 (9.0)	104 (19.6)	
Pneumothorax requires treatment, n(%)			0.30			0.25
No	696 (98.9)	711 (98.2)		525 (98.7)	520 (97.7)	
Yes	8 (1.1)	13 (1.8)		7 (1.3)	12 (2.3)	
Cardiac complication, n(%)			**0.02**			0.22
No	689 (97.9)	692 (95.6)		519 (97.6)	512 (96.2)	
Yes	15 (2.1)	32 (4.4)		13 (2.4)	20 (3.8)	
Anastomotic leak, n(%)			0.19			0.91
No	647 (91.9)	651 (89.9)		486 (91.3)	487 (91.5)	
Yes	57 (8.1)	73 (10.1)		46 (8.7)	45 (8.5)	
Recurrent laryngeal nerve palsy, n(%)			**0.04**			**0.04**
No	573 (81.4)	619 (85.5)		426 (80.1)	451 (84.8)	
Yes	131 (18.6)	105 (14.5)		106 (19.9)	81 (15.2)	
Wound infections, n(%)			1.0			1.0
No	703 (99.9)	722 (99.7)		531 (99.8)	530 (99.6)	
Yes	1 (0.1)	2 (0.3)		1 (0.2)	2 (0.4)	
Chylothorax, n(%)			0.21			0.54
No	682 (96.9)	709 (97.9)		518 (97.4)	521 (97.9)	
Yes	22 (3.1)	15 (2.1)		14 (2.6)	11 (2.1)	
Re-operation, n(%)			0.97			0.62
No	700 (99.4)	721 (99.6)		529 (99.44)	531 (99.81)	
Yes	4 (0.6)	3 (0.4)		3 (0.56)	1 (0.19)	
90-d hospital mortality, n(%)			0.35			0.62
No	700 (99.4)	723 (99.9)		529 (99.4)	531 (99.8)	
Yes	4 (0.6)	1 (0.1)		3 (0.6)	1 (0.2)	

© 2025 Guo X et al. *JAMA Surgery.*

eTable 3. Recurrence patterns of postoperative two years between the NCRT group and the NCIT group before and after propensity score matching.

	Before PSM			After PSM		
	NCRT (n = 704)	NCIT (n = 724)	*P*	NCRT (n = 532)	NCIT (n = 532)	*P*
**Recurrence**			**<.001**			**<.001**
No	451 (64.1)	560 (77.4)		342 (64.3)	406 (76.3)	
Yes	253 (35.9)	164 (22.6)		190 (35.7)	126 (23.7)	
**Recurrence pattern**						
Localregional recurrence, n(%)			0.14			0.50
No	554 (78.7)	598 (82.6)		421 (79.1)	434 (81.6)	
Yes	150(21.3)	126(17.4)		111(20.9)	98(18.4)	
Lymph nodes	117 (16.6)	101 (13.9)		86 (16.2)	81 (15.2)	
Anastomosis	18 (2.6)	18 (2.5)		15 (2.8)	12 (2.3)	
Anastomosis and lymph nodes	15 (2.1)	7 (1.0)		10 (1.9)	5 (0.9)	
Distant metastasis, n(%)			**<.001**			**<.001**
No	515 (73.1)	626 (86.5)		399 (75.0)	460 (86.5)	
Yes	189 (26.9)	98 (13.5)		133 (25.0)	72 (13.5)	
Bone, n(%)			**<.001**			**<.001**
No	650 (92.3)	702 (97.0)		487 (91.5)	517 (97.2)	
Yes	54 (7.7)	22 (3.0)		45 (8.5)	15 (2.8)	
Liver, n(%)			**0.006**			0.06
No	643 (91.3)	688 (95.0)		490 (92.1)	505 (94.9)	
Yes	61 (8.7)	36 (5.0)		42 (7.9)	27 (5.1)	
Lung, n(%)			**<.001**			**<.001**
No	617 (87.6)	693 (95.7)		472 (88.7)	506 (95.1)	
Yes	87 (12.4)	31 (4.3)		60 (11.3)	26 (4.9)	
Pleura/Pericardium/Perioneum, n(%)			**0.004**			**0.007**
No	682 (96.9)	717 (99.0)		516 (97.0)	528 (99.2)	
Yes	22 (3.1)	7 (1.0)		16 (3.0)	4 (0.8)	
Brain, n(%)			0.80			0.65
No	687 (97.6)	708 (97.8)		521 (97.9)	523 (98.3)	
Yes	17 (2.4)	16 (2.2)		11 (2.1)	9 (1.7)	
Pancreas, n(%)			0.71			1.000
No	702 (99.7)	720 (99.4)		530 (99.6)	529 (99.4)	
Yes	2 (0.3)	4 (0.6)		2 (0.4)	3 (0.6)	
Kidney, n(%)			0.14			0.48
No	704 (100.0)	720 (99.5)		532 (100.0)	530 (99.6)	
Yes	0 (0.0)	4 (0.6)		0 (0.0)	2 (0.4)	
Adrenal gland, n(%)			0.98			1.0
No	701 (99.6)	722 (99.7)		530 (99.6)	531 (99.8)	
Yes	3 (0.4)	2 (0.3)		2 (0.4)	1 (0.2)	
104LN, n(%)			0.45			0.40
No	693 (98.4)	716 (98.9)		524 (98.5)	527 (99.1)	
Yes	11 (1.6)	8 (1.1)		8 (1.5)	5 (0.9)	
Combined recurrence			**0.032**			0.50
No	658(93.5)	695(96.0)		503(94.5)	508(95.5)	
Yes	46(6.5)	29(4.0)		29(5.5)	24(4.5)	

© 2025 Guo X et al. *JAMA Surgery.*

eTable 4. Recurrence patterns of MPR and Non-MPR between the NCRT group and the NCIT group before and after propensity score matching.

	Before PSM						After PSM					
	MPR			Non-MPR			MPR			Non-MPR		
	NCRT (n = 518)	NCIT (n = 444)	*P*	NCRT (n = 186)	NCIT (n = 280)	*P*	NCRT (n = 382)	NCIT (n = 327)	*P*	NCRT (n = 150)	NCIT (n = 205)	*P*
Localregional recurrence, n(%)			0.19			0.139			0.80			0.29
None	426 (82.3)	383 (86.3)		128 (68.8)	215 (76.8)		319 (83.5)	279 (85.4)		102 (68.0)	155 (75.6)	
Lymph nodes	76 (14.7)	49 (11.0)		41 (22.0)	52 (18.6)		52 (13.6)	41 (12.5)		34 (22.7)	40 (19.5)	
Anastomosis	8 (1.5)	9 (2.0)		10 (5.4)	9 (3.2)		6 (1.6)	5 (1.5)		9 (6.0)	7 (3.4)	
Anastomosis and lymph nodes	8 (1.5)	3 (0.7)		7 (3.8)	4 (1.4)		5 (1.3)	2 (0.6)		5 (3.3)	3 (1.5)	
Distant metastasis, n(%)			**<.001**			**<.001**			**<.001**			**0.002**
No	400 (77.2)	403 (90.8)		115 (61.8)	223 (79.6)		306 (80.1)	302 (92.4)		93 (62.0)	158 (77.1)	
Yes	118 (22.8)	41 (9.2)		71 (38.2)	57 (20.4)		76 (19.9)	25 (7.6)		57 (38.0)	47 (22.9)	
Bone, n(%)			**<.001**			**0.02**			**<.001**			**0.01**
No	483 (93.2)	435 (98.0)		167 (89.8)	267 (95.4)		355 (92.9)	322 (98.5)		132 (88.0)	195 (95.1)	
Yes	35 (6.8)	9 (2.0)		19 (10.2)	13 (4.6)		27 (7.1)	5 (1.5)		18 (12.0)	10 (4.9)	
Liver, n(%)			**0.003**			0.10			**0.02**			0.31
No	481 (92.9)	431 (97.1)		162 (87.1)	257 (91.8)		359 (94.0)	319 (97.8)		131 (87.3)	186 (90.7)	
Yes	37 (7.1)	13 (2.9)		24 (12.9)	23 (8.2)		23 (6.0)	8 (2.2)		19 (12.7)	19 (9.3)	
Lung, n(%)			**<.001**			**<.001**			**0.02**			**<.001**
No	471 (90.9)	428 (96.4)		146 (78.5)	265 (94.6)		353 (92.4)	316 (96.6)		119 (79.3)	190 (92.7)	
Yes	47 (9.1)	16 (3.6)		40 (21.5)	15 (5.4)		29 (7.6)	11 (3.4)		31 (20.7)	15 (7.3)	
Pleura/Pericardium/Perioneum, n(%)			**<.001**			0.95			**0.001**			0.93
No	501 (96.7)	443 (99.8)		181 (97.3)	274 (97.9)		370 (96.9)	327 (100.0)		146 (97.3)	201 (98.0)	
Yes	17 (3.3)	1 (0.2)		5 (2.7)	6 (2.1)		12 (3.1)	0 (0.0)		4 (2.7)	4 (2.0)	
Brain, n(%)			0.41			0.75			0.48			1.0
No	506 (97.7)	437 (98.4)		181 (97.3)	271 (96.8)		375 (98.2)	324 (99.1)		146 (97.3)	199 (97.1)	
Yes	12 (2.3)	7 (1.6)		5 (2.7)	9 (3.2)		7 (1.8)	3 (0.9)		4 (2.7)	6 (2.9)	
Pancreas, n(%)			1.0			0.92			1.0			0.85
No	517 (99.8)	443 (99.8)		185 (99.5)	277 (98.9)		381 (99.7)	327 (100.0)		149 (99.3)	202 (98.5)	
Yes	1 (0.2)	1 (0.2)		1 (0.5)	3 (1.1)		1 (0.3)	0 (0.0)		1 (0.7)	3 (1.5)	
Kidney, n(%)			0.46			0.41			1.0			0.51
No	518 (100.0)	443 (99.8)		186 (100.0)	277 (98.9)		382 (100.0)	327(100.0)		150 (100.0)	203 (99.0)	
Yes	0 (0.0)	1 (0.2)		0 (0.0)	3 (1.1)		0 (0.0)	0 (0.0)		0 (0.0)	2 (1.0)	
Adrenal gland, n(%)			1.0			1.0			1.0			1.0
No	516 (99.6)	443 (99.8)		185 (99.5)	279 (99.6)		381 (99.7)	327 (100.0)		149 (99.3)	204 (99.5)	
Yes	2 (0.4)	1 (0.2)		1 (0.5)	1 (0.4)		1 (0.3)	0 (0.0)		1 (0.7)	1 (0.5)	
104LN, n(%)			0.27			1.0			0.58			1.0
No	511 (98.7)	442 (99.5)		182 (97.9)	274 (97.9)		377 (98.7)	325 (99.4)		147 (98.0)	202 (98.5)	
Yes	7 (1.3)	2 (0.5)		4 (2.1)	6 (2.1)		5 (1.3)	2 (0.6)		3 (2.0)	3 (1.5)	

© 2025 Guo X et al. *JAMA Surgery.*

eTable 5. Recurrence patterns of pCR and Non-pCR between the NCRT group and the NCIT group before and after propensity score matching.

	Before PSM						After PSM					
	pCR			Non-pCR			pCR			Non-pCR		
	NCRT (n = 180)	NCIT (n = 164)	*P*	NCRT (n = 524)	NCIT (n = 560)	*P*	NCRT (n = 138)	NCIT (n = 122)	*P*	NCRT (n = 394)	NCIT (n = 410)	*P*
Localregional recurrence, n(%)			0.17			0.38			0.62			0.67
None	156 (86.7)	150 (91.5)		398 (76.0)	448 (80.0)		119 (86.2)	110 (90.2)		302 (76.7)	324 (79.0)	
Lymph nodes	18 (10.0)	11 (6.7)		99 (18.9)	90 (16.1)		15 (11.0)	10 (8.2)		71 (18.0)	71 (17.3)	
Anastomosis	2 (1.1)	3 (1.8)		16 (3.0)	15 (2.7)		2 (1.4)	2 (1.6)		13 (3.3)	10 (2.5)	
Anastomosis and lymph nodes	4 (2.2)	0 (0.0)		11 (2.1)	7 (1.2)		2 (1.4)	0 (0.0)		8 (2.0)	5 (1.2)	
Distant metastasis, n(%)			**0.002**			**<.001**			**0.02**			**<.001**
No	148 (82.2)	153 (93.3)		367 (70.0)	473 (84.5)		120 (87.0)	116 (95.1)		279 (70.8)	344 (83.9)	
Yes	32 (17.8)	11 (6.7)		157 (30.0)	87 (15.5)		18 (13.0)	6 (4.9)		115 (29.2)	66 (16.1)	
Bone, n(%)			0.11			**<.001**			0.37			**<.001**
No	171 (95.0)	161 (98.2)		479 (91.4)	541 (96.6)		132 (95.6)	120 (98.4)		355 (90.1)	397 (96.8)	
Yes	9 (5.0)	3 (1.8)		45 (8.6)	19 (3.4)		6 (4.4)	2 (1.6)		39 (9.9)	13 (3.2)	
Liver, n(%)			0.10			**0.02**			0.44			0.08
No	173 (96.1)	163 (99.4)		470 (89.7)	525 (93.7)		134 (97.1)	121 (99.2)		356 (90.4)	384 (93.7)	
Yes	7 (3.9)	1 (0.6)		54 (10.3)	35 (6.3)		4 (2.9)	1 (0.8)		38 (9.6)	26 (6.3)	
Lung, n(%)			**0.03**			**<.001**			0.44			**<.001**
No	166 (92.2)	160 (97.6)		451 (86.1)	533 (95.2)		131 (94.9)	119 (97.5)		341 (86.5)	387 (94.4)	
Yes	14 (7.8)	4 (2.4)		73 (13.9)	27 (4.8)		7 (5.1)	3 (2.5)		53 (13.5)	23 (5.6)	
Pleura/Pericardium/Perioneum, n(%)			**0.03**			0.06			0.10			0.06
No	173 (96.1)	164 (100.0)		509 (97.1)	553 (98.7)		133 (96.4)	122 (100.0)		383 (97.2)	406 (99.0)	
Yes	7 (3.9)	0 (0.0)		15 (2.9)	7 (1.3)		5 (3.6)	0 (0.0)		11 (2.8)	4 (1.0)	
Brain, n(%)			0.60			0.45			0.47			0.43
No	178 (98.9)	160 (97.6)		509 (97.1)	548 (97.9)		138 (100.0)	121 (99.2)		383 (97.2)	402 (98.0)	
Yes	2 (1.1)	4 (2.4)		15 (2.9)	12 (2.1)		0 (0.0)	1 (0.8)		11 (2.8)	8 (2.0)	
Pancreas, n(%)			1.0			0.66			1.0			0.64
No	179 (99.4)	163 (99.4)		523 (99.8)	557 (99.5)		137 (99.3)	122 (100.0)		393 (99.7)	407 (99.3)	
Yes	1 (0.6)	1 (0.6)		1 (0.2)	3 (0.5)		1 (0.7)	0 (0.0)		1 (0.3)	3 (0.7)	
Kidney, n(%)			1.0			0.15			1.0			0.50
No	180 (100.0)	164 (100.0)		524 (100.0)	556 (99.3)		138 (100.0)	122 (100.0)		394 (100.0)	408 (99.5)	
Yes	0 (0.0)	0 (0.0)		0 (0.0)	4 (0.7)		0 (0.0)	0 (0.0)		0 (0.0)	2 (0.5)	
Adrenal gland, n(%)			1.0			1.0			1.0			0.97
No	179 (99.4)	164 (100.0)		522 (99.6)	558 (99.6)		138 (100.0)	122(100.0)		392 (99.5)	409 (99.8)	
Yes	1 (0.6	0 (0.0)		2 (0.4	2 (0.4)		0 (0.0)	0 (0.0)		2 (0.51)	1 (0.2)	
104LN, n(%)			1.0			0.54			1.0			0.36
No	179 (99.4)	164 (100.0)		514 (98.1)	552 (98.6)		138 (100.0)	122 (100.0)		386 (98.0)	405 (98.8)	
Yes	1 (0.6	0 (0.0)		10 (1.9)	8 (1.4)		0 (0.0)	0 (0.0)		8 (2.0)	5 (1.2)	

eFigure 1: A scatter plot of the absolute mean differences in baseline data between the NCRT and NCIT groups before and after matching

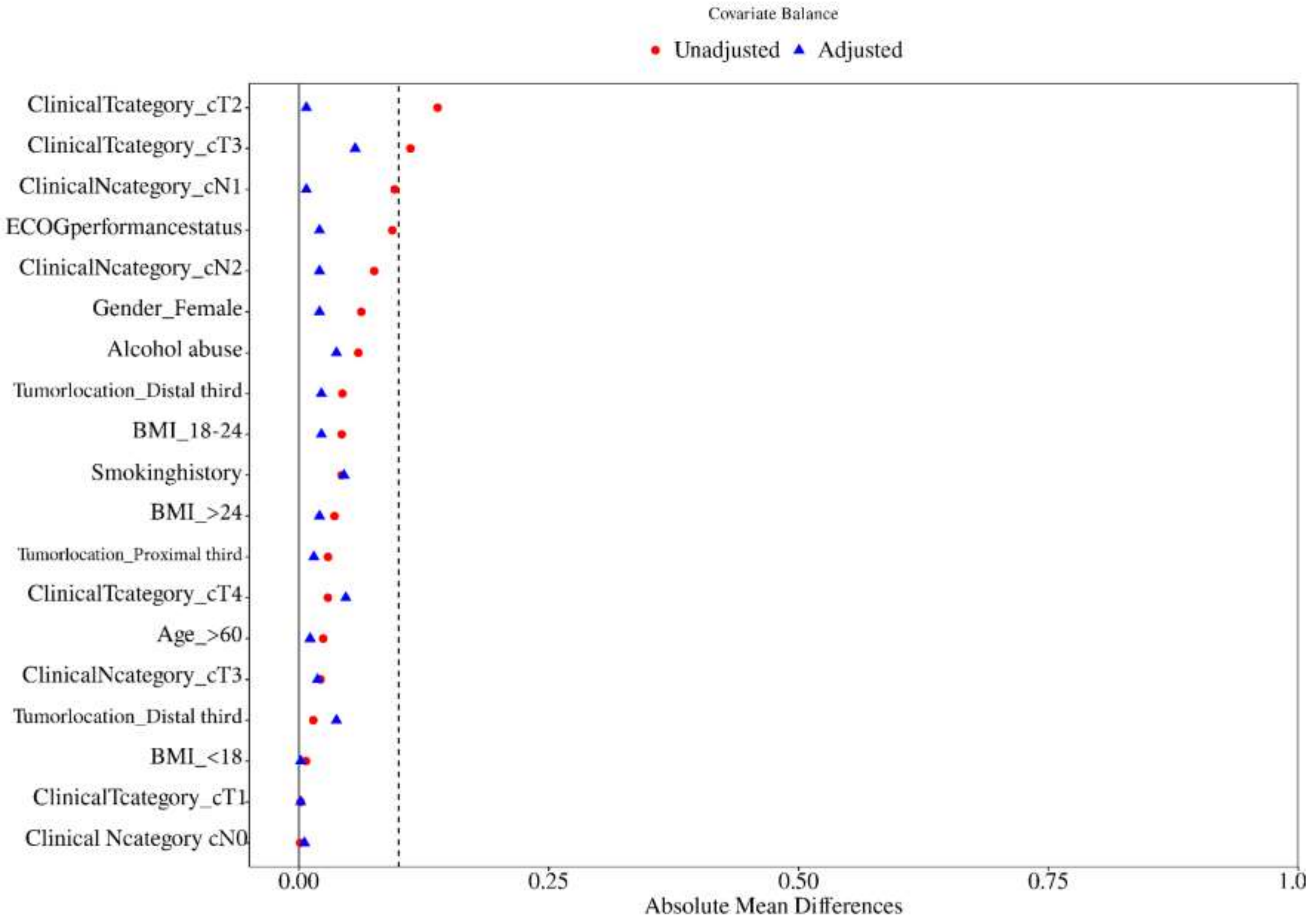

Red circles: before matching; Blue triangles: after matching

© 2025 Guo X et al. *JAMA Surgery.*

eFigure 2: Subgroup analysis of disease-free survival and overall survival were conducted between the NCIT and NCRT groups, stratified by MPR or Non-MPR

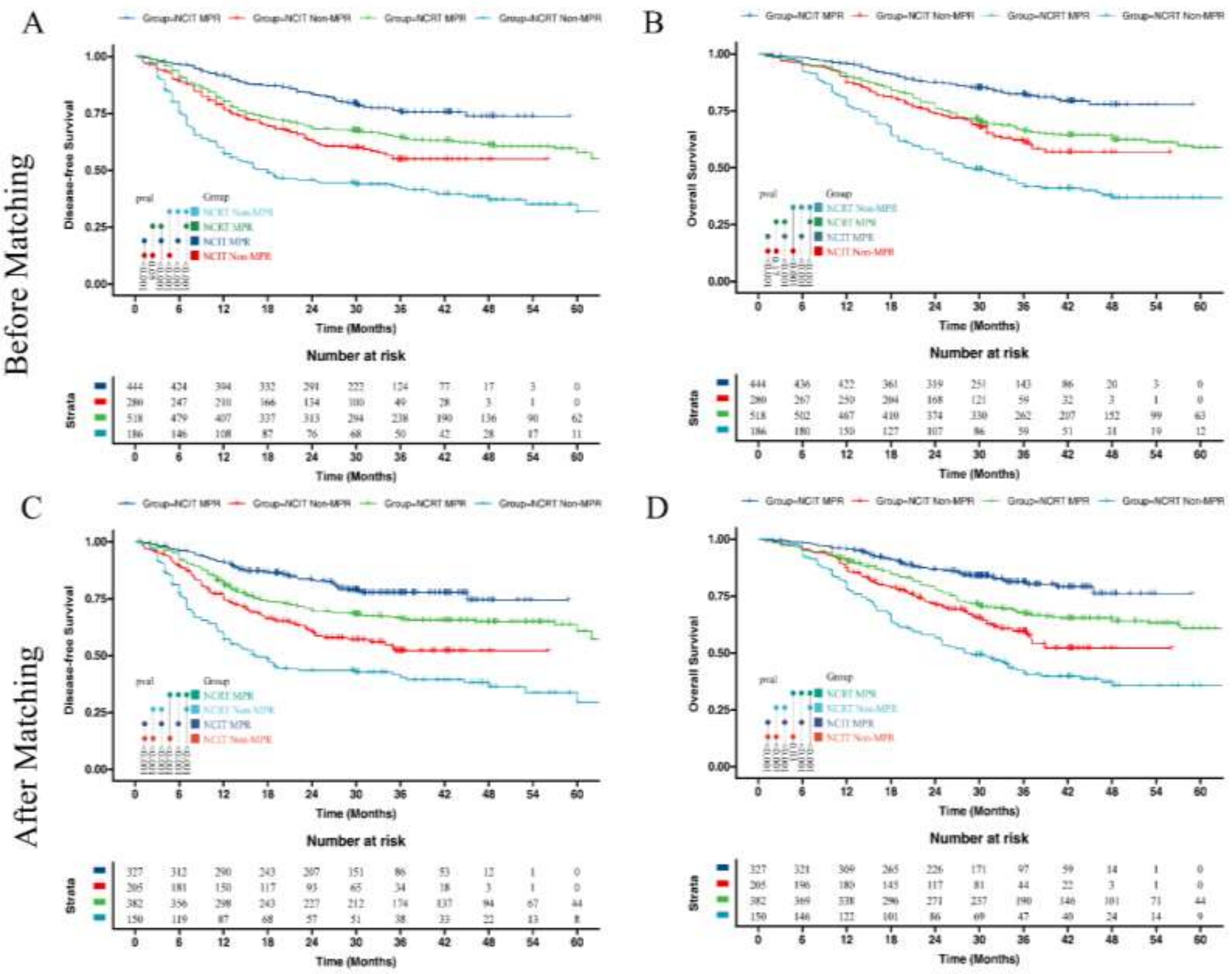

Before matching: (A) Disease-free survival (B) Overall survival.

After matching: (C) Disease-free survival (D) Overall survival.HR=hazard ratio.

© 2025 Guo X et al. *JAMA Surgery.*

eFigure 3: Subgroup analysis of disease-free survival and overall survival were conducted between the NCIT and NCRT groups, stratified by pCR or Non-pCR

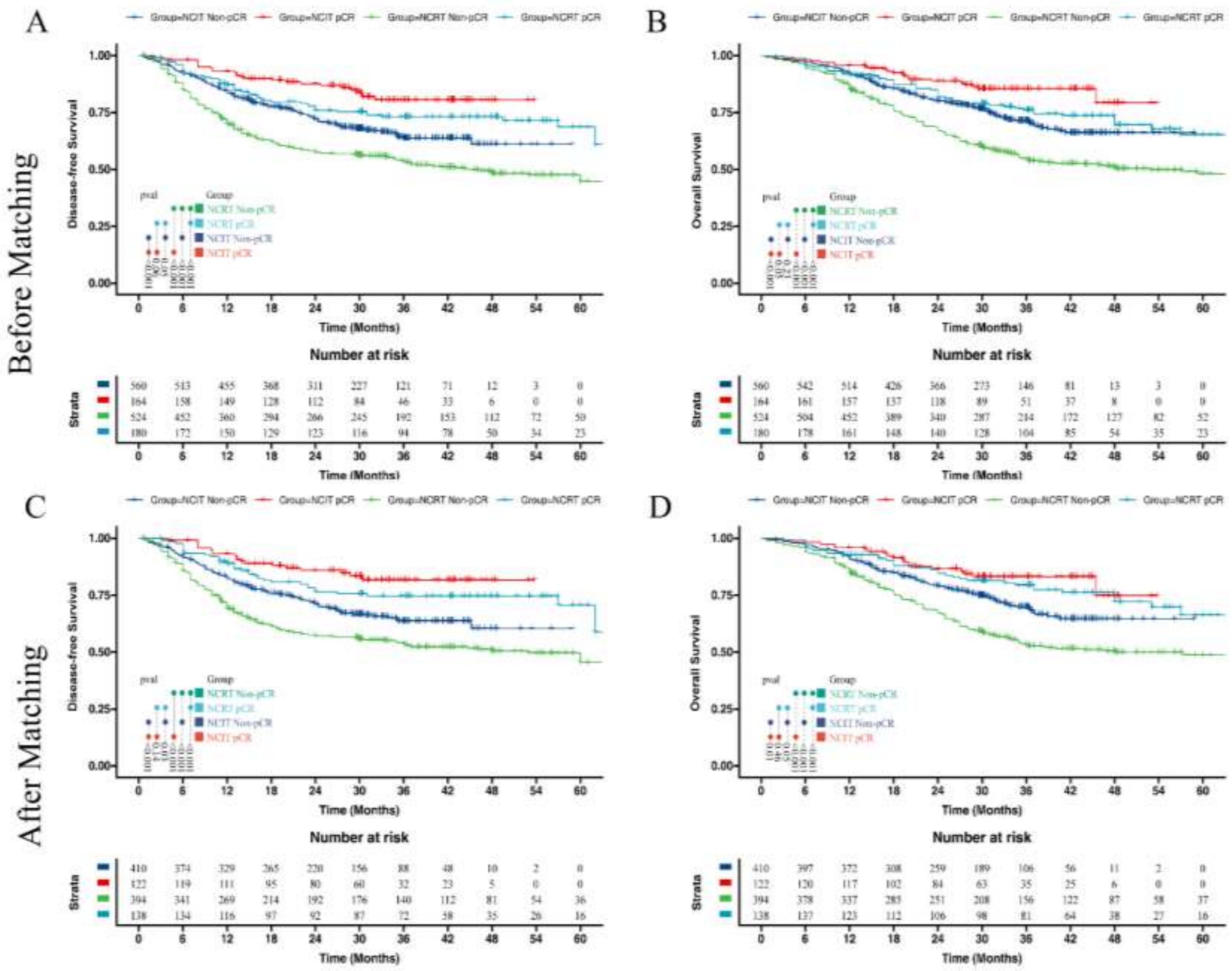

Before matching: (A) Disease-free survival (B) Overall survival.

After matching: (C) Disease-free survival (D) Overall survival.HR=hazard ratio.

© 2025 Guo X et al. *JAMA Surgery.*

eFigure 4: Kaplan-Meier estimates of locoregional relapse-free survival (LRFS) and distant metastasis-free survival (DMFS) between NCIT and NCRT

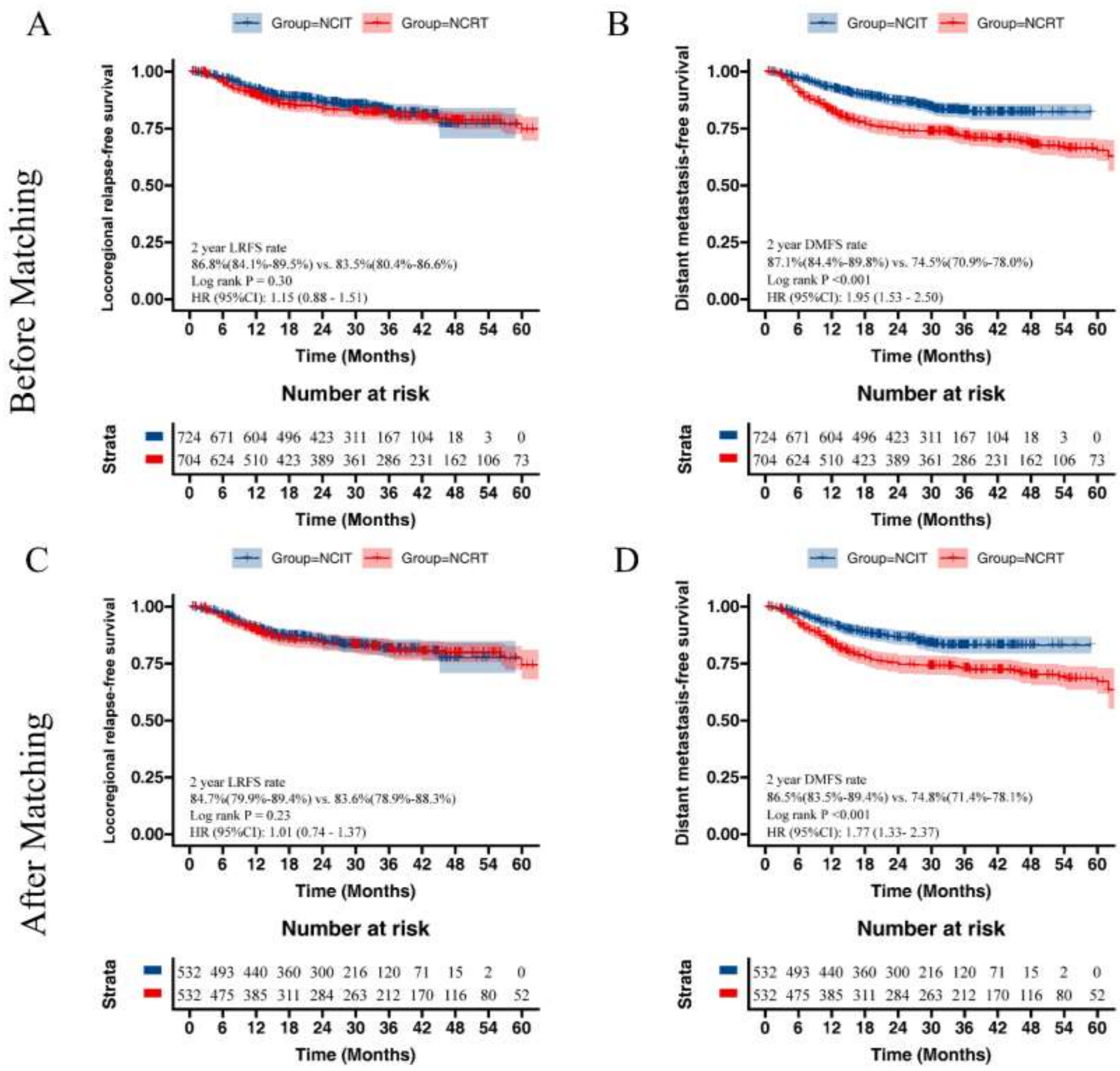

Before matching: (A) LRFS (B) DMFS.

After matching: (C) LRFS (D) DMFS.HR=hazard ratio.

© 2025 Guo X et al. *JAMA Surgery.*

eFigure 5: Subgroup analyses of disease-free survival and overall survival between the NCRT group and NCIT group (stratified by adjuvant immunotherapy)

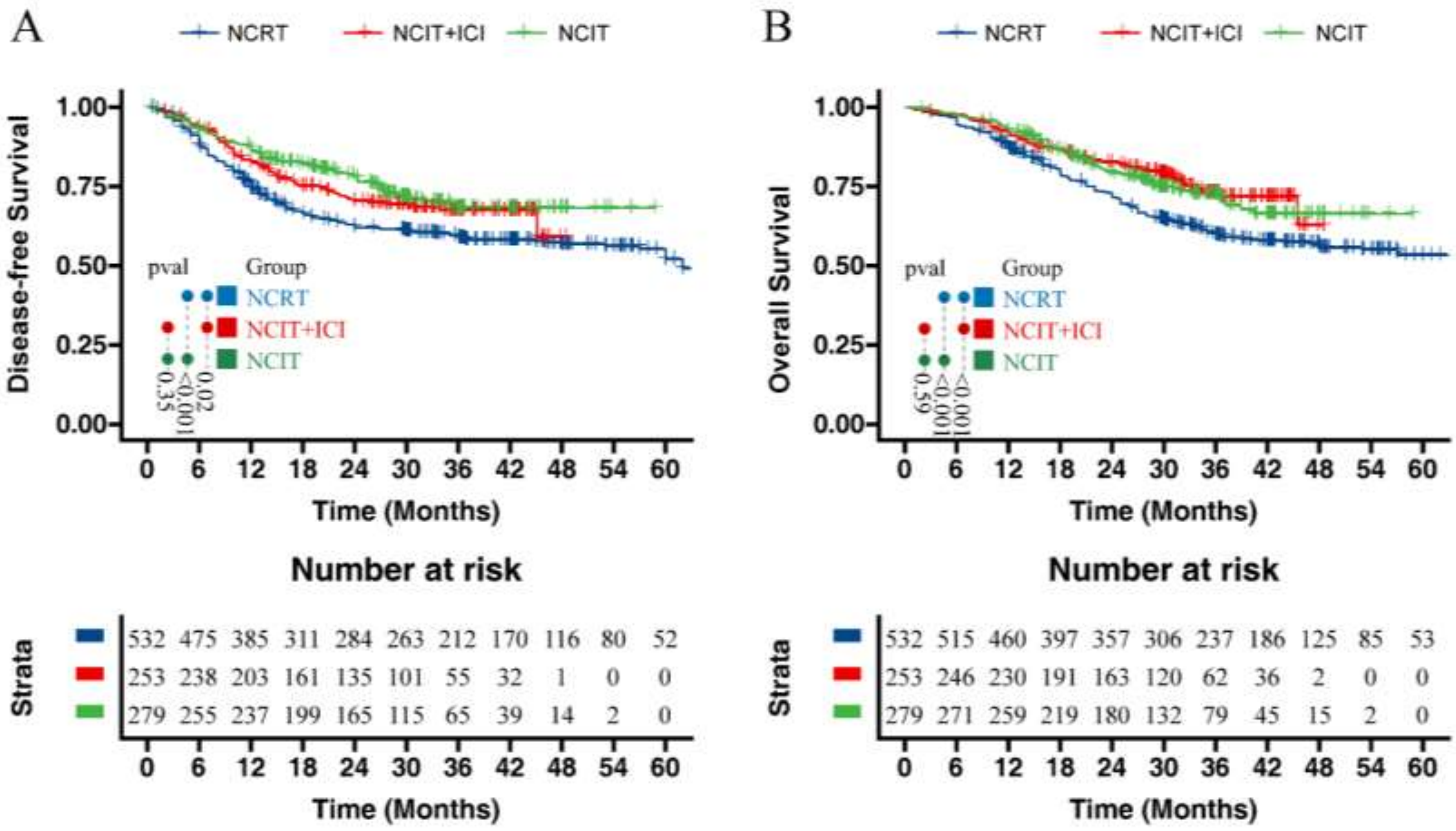

(A) Disease-free survival (B) Overall survival.

© 2025 Guo X et al. *JAMA Surgery.*
