# Supplementary material for: Neoadjuvant Chemoradiotherapy vs Chemoimmunotherapy for Esophageal Squamous Cell Carcinoma
Source: JAMA Surg. 2025 Mar 19;160(5):565–74. doi: 10.1001/jamasurg.2025.0220 (PMC11923775; doi:10.1001/jamasurg.2025.0220)
Supplement: Supplement 2. — Data Sharing Statement [file jamasurg-e250220-s002.pdf]

Data Sharing Statement

Guo. Neoadjuvant Chemoradiotherapy vs Chemoimmunotherapy for Esophageal Squamous Cell Carcinoma. *JAMA Surg*. Published March 19, 2025. doi:10.1001/jamasurg.2025.0220

Data
Data available: Yes
Data types: Deidentified participant data
How to access data: De-identified individual participant data, along with the study protocol, statistical analysis plan, and informed consent form, will be made available upon reasonable request to the corresponding author, following publication. Data will be available for a period of 6 months following publication, for the purposes of academic research only, and after the approval of a methodologically sound research proposal. Requests should be directed to Zhigang Li, Email: zhigang.li@shsmu.edu.cn.
When available: With publication

Supporting Documents
Document types: None

Additional Information
Who can access the data: researchers whose proposed use of the data has been approved
Types of analyses: for the purposes of academic research only
Mechanisms of data availability: with investigator support
